# Improving Compliance With Surgical Scrubbing and Sterilization Protocols: A Two-Cycle Quality Improvement Project at Atbara Teaching Hospital

**DOI:** 10.7759/cureus.112559

**Published:** 2026-07-13

**Authors:** Abubaker Mahmood Mohamed Osman, Saifalden Mustafa Badralden Mustafa, Ahmed Awadelkareem Omer Mahmoud, Baligh Mohammed Salih Kubashi, Mohammed Abdelmagid Yousif Ahmed, Aya Mustafa Ahmed Mohamed, Mahmoud Essamayed Mohamed Badawy, Ahmed Abdelmoneim Gameel Mustafa, Algazali Abdelrahman Ahmed Jadalla, Hussein Abdelaziz Suliman Ahmed, Hozifa Hussein Abdelgayoum Mohamed, Lamees Adil Abdulbaqi Zeinalabedeen, Manal Nasreldeen Mohamed Wahab, Mohammed Ahmed, Azza Ali Osman Mohammed, Abdelazeem Bakhite Adam Nassour, Aseel Ibrahim Musa Osman, Adam Elnour Mohamed Ibrahim

**Affiliations:** 1 Surgery, Atbara Teaching Hospital, Atbara, SDN; 2 Pediatrics, Atbara Teaching Hospital, Atbara , SDN; 3 Quality Administration, Atbara Teaching Hospital, Atbara , SDN; 4 General Medicine, Brownlow Health, Liverpool, GBR; 5 Pediatrics and Child Health, Sudan International University, Khartoum, SDN; 6 Plastic and Reconstructive Surgery, Atbara Teaching Hospital, Atbara, SDN; 7 General Physician, Karary University, Khartoum, SDN; 8 Medicine, University of Al Fashir, Al Fashir, SDN

**Keywords:** aseptic technique, clinical audit, hand hygiene, infection prevention, operating theatre, pdsa cycle, quality improvement, sterilization, surgical scrubbing, surgical site infection

## Abstract

Background

Surgical site infections remain a major cause of postoperative morbidity, prolonged hospitalization, increased healthcare costs, and preventable mortality worldwide. Adherence to evidence-based surgical scrubbing, sterilization, and aseptic practices is essential for reducing contamination risks and maintaining patient safety within operating theatres. This quality improvement project aimed to assess compliance with internationally recommended surgical scrubbing and sterilization standards, implement targeted interventions, and evaluate their impact using a closed-loop audit methodology.

Methods

A prospective closed-loop clinical audit was conducted in the operating theatre complex of Atbara Teaching Hospital, Sudan, using the Plan-Do-Study-Act (PDSA) framework. Twenty-seven evidence-based criteria derived from international infection prevention guidelines were evaluated across five domains: surgical attire, surgical hand hygiene and scrubbing, gowning and gloving, anesthesia-related infection prevention, and sterile field preparation. The first audit cycle was conducted between March and May 2024, followed by a multifaceted intervention consisting of education, audit feedback, visual reminders, and multidisciplinary engagement. A re-audit was subsequently performed between January and May 2025 using the same methodology and assessment criteria. Compliance rates were compared between audit cycles using Pearson’s Chi-square test or Fisher’s exact test where appropriate.

Results

A total of 29 surgical procedures were observed during the first audit cycle and 25 during the second cycle. Following implementation of the intervention package, significant improvements were observed across all assessed domains. Compliance with head-cover use increased from 47.7% to 70.7%, surgical scrubbing before gowning or gloving improved from 50.0% to 88.2%, correct gowning procedures increased from 44.4% to 91.3%, anesthesiologist hand hygiene improved from 22.2% to 69.6%, and adherence to the correct sequence for opening sterile items increased from 44.4% to 95.7%. All evaluated domains demonstrated statistically significant improvements following the intervention (p<0.05).

Conclusion

Implementation of a multifaceted quality improvement intervention was associated with substantial improvements in compliance with surgical scrubbing, sterilization, and infection prevention standards within the operating theatre. These findings support the effectiveness of structured clinical audit, audit feedback, and targeted educational strategies in improving adherence to evidence-based perioperative infection prevention practices, which may contribute to improved patient safety in surgical settings.

## Introduction

Surgical site infections (SSIs) remain among the most common healthcare-associated infections worldwide and continue to represent a major cause of preventable postoperative morbidity, prolonged hospitalization, increased healthcare expenditure, and mortality. Despite significant advances in surgical techniques and perioperative care, SSIs continue to affect millions of patients annually, particularly in low- and middle-income countries (LMICs) where resource limitations, overcrowding, and inconsistencies in infection prevention practices may increase the risk of healthcare-associated infections. The World Health Organization (WHO) estimates that SSIs affect approximately 11% of surgical patients in low- and middle-income settings, with substantially higher rates reported in some African healthcare facilities. Effective prevention of SSIs therefore remains a critical global patient safety priority [1].

Strict adherence to surgical scrubbing, sterile attire, hand antisepsis, gowning, gloving, and operating room sterilization protocols constitutes one of the most important components of infection prevention and control within surgical environments. Proper surgical hand preparation reduces microbial burden on the hands of healthcare workers and minimizes the risk of contamination of the sterile field, particularly in situations involving unnoticed glove perforations during surgical procedures. Evidence demonstrates that contamination originating from surgical personnel remains an important contributor to perioperative microbial transmission and subsequent postoperative infections [2].

International guidelines emphasize the importance of maintaining aseptic practices throughout all phases of surgical care. The WHO Global Guidelines for the Prevention of Surgical Site Infection recommend surgical hand preparation using either antimicrobial soap and water or alcohol-based hand rub formulations before donning sterile gloves, alongside strict adherence to perioperative infection prevention measures [3]. Similarly, the Association of periOperative Registered Nurses (AORN) identifies surgical attire, hand hygiene, sterile technique, and appropriate gowning and gloving practices as essential evidence-based measures for reducing microbial contamination and promoting patient safety in operating rooms [4].

Surgical scrubbing and sterilization practices are well established and standardized through internationally recognized infection prevention guidelines. Nevertheless, studies from diverse healthcare settings have consistently reported variable adherence to these standards, with deficiencies including inadequate hand hygiene, improper use of surgical attire, failure to replace contaminated protective equipment, inappropriate jewelry use, and breaches in sterile field maintenance. Such lapses have been associated with increased contamination risks and compromised infection prevention practices. Consequently, clinical audit and quality improvement initiatives have become increasingly recognized as effective strategies for identifying implementation gaps, monitoring adherence to established standards, and promoting sustainable improvements in perioperative practice among surgical teams [5,6].

Quality improvement methodologies, particularly the Plan-Do-Study-Act (PDSA) framework, provide a structured approach for evaluating current practices, implementing targeted interventions, and measuring improvements over time. Previous evidence suggests that educational programs, visual reminders, audit-feedback systems, and multidisciplinary engagement can significantly enhance compliance with infection prevention standards and contribute to safer perioperative environments [7].

Atbara Teaching Hospital is one of the major referral hospitals in River Nile State, Sudan, where surgical services are provided across multiple specialties. Although studies from LMICs have reported variable compliance with surgical scrubbing and sterilization standards and highlighted the benefits of clinical audit for improving adherence to infection prevention practices, comparable audits remain limited in Sudan. Furthermore, to the best of our knowledge, no previous comprehensive audit has systematically evaluated compliance with surgical scrubbing and sterilization standards among different categories of operating room personnel at Atbara Teaching Hospital. This quality improvement project was therefore conducted to assess baseline adherence to internationally recommended scrubbing and sterilization practices, implement targeted improvement interventions, and evaluate the effectiveness of these interventions through a two-cycle audit using the PDSA methodology.

## Materials and methods

Study design

This quality improvement project employed a prospective closed-loop clinical audit design using the PDSA methodology to evaluate and improve compliance with surgical scrubbing and sterilization practices within the operating theatres of Atbara Teaching Hospital, River Nile State, Sudan. The project consisted of two audit cycles separated by a structured intervention phase. Baseline performance was assessed during the first audit cycle, after which targeted improvement measures were implemented and subsequently evaluated during the second audit cycle.

Study setting

The project was conducted in the operating theatre complex of Atbara Teaching Hospital, a major tertiary referral and teaching institution serving River Nile State and surrounding regions of Sudan. The hospital performs a wide range of elective and emergency surgical procedures across multiple specialties and functions as an important training center for surgeons, anesthesiologists, nurses, and other healthcare professionals involved in perioperative care.

Audit standards and criteria

Audit standards were derived from internationally recognized infection prevention and perioperative safety guidelines, including the WHO Guidelines for the Prevention of Surgical Site Infection, WHO recommendations on surgical hand preparation, the Association of Surgical Technologists (AST) Standards of Practice for Surgical Attire, Surgical Scrub, Hand Hygiene and Hand Washing, AST recommendations for establishing and maintaining the sterile field, and guidance from the International Federation of Perioperative Nurses regarding surgical hand scrubbing and aseptic practice [1,8].

All eligible surgical procedures performed during the predefined audit periods were included consecutively using a census sampling approach, consistent with recommended clinical audit methodology. Surgical procedures involving operating theatre personnel responsible for surgical scrubbing, gowning, gloving, and sterile field maintenance were eligible for inclusion. Procedures were excluded if direct observation could not be completed for the entire perioperative period or if key operating room personnel were unavailable for assessment. This consecutive sampling approach was adopted to minimize selection bias and provide a representative assessment of routine clinical practice.

A comprehensive audit tool consisting of 27 evidence-based criteria was developed from these standards. The criteria were grouped into five principal domains: 1) Surgical attire and clothing before entering the restricted operating room zone; 2) Surgical hand hygiene, hand scrubbing, and sterilization practices; 3) Gowning and gloving procedures after entering the operating room; 4) Anesthesia-related infection prevention and sterilization practices; 5) Sterile field preparation and management of sterile supplies.

Each criterion was assessed as compliant or non-compliant according to predefined operational definitions based on the referenced international standards.

Study population

The audit evaluated members of the multidisciplinary surgical team participating in operative procedures during the study periods. Observations included surgeons, assistant surgeons, scrubbing nurses, circulating nurses, anesthesiologists, and trainers when present during procedures.

A total of 29 surgical procedures were observed during the first audit cycle and 25 procedures during the second audit cycle. During each procedure, compliance with the applicable audit criteria was assessed among the relevant members of the operating theatre team. Because multiple professional groups could be observed within a single procedure, each operation generated several observation opportunities for assessment.

Audit cycles

First Audit Cycle

The baseline audit cycle was conducted between March 12, 2024 and May 31, 2024. During this phase, direct observations of operating theatre practices were performed using standardized data collection tools. Compliance with all predefined audit criteria was measured to establish baseline performance and identify areas requiring improvement.

Intervention Phase

Following completion of the baseline assessment, audit findings were analyzed and presented to relevant stakeholders, including surgeons, assistant surgeons, scrub nurses, circulating nurses, anesthesiologists, infection prevention representatives, and hospital administrators. Baseline compliance rates, identified deficiencies, and priority areas for improvement were discussed during multidisciplinary feedback meetings.

A multifaceted improvement strategy was subsequently implemented. Educational sessions were delivered to operating theatre personnel and focused on evidence-based surgical hand preparation, surgical attire, gowning and gloving techniques, maintenance of the sterile field, aseptic practice, and sterilization procedures in accordance with internationally recognized infection prevention guidelines. Audit findings and identified practice gaps were shared with staff, followed by targeted feedback and discussion of corrective measures. Visual reminder materials, including educational posters summarizing key infection prevention practices, were displayed at hand-scrubbing stations and other prominent areas within the operating theatre to reinforce recommended practices. Multidisciplinary engagement was promoted by encouraging active participation of all operating theatre personnel in reviewing audit findings, discussing improvement strategies, and supporting adherence to standardized perioperative infection prevention practices.

The intervention package was developed using the PDSA framework to address deficiencies identified during the baseline audit, reinforce evidence-based practice, and promote sustainable improvements in compliance among operating theatre personnel.

Second Audit Cycle

The re-audit cycle was conducted between January 1, 2025 and May 31, 2025 using the same audit criteria, methodology, and data collection instruments employed during the baseline assessment. This ensured direct comparability between the two audit cycles and allowed evaluation of the effectiveness of the implemented interventions.

Data collection

Data were collected through overt, direct non-participant observation of surgical procedures within the operating theatre environment using structured audit checklists developed specifically for the project. The audit was conducted by five trained observers, comprising one specialist, two registrars, and two medical officers, who assessed compliance with predefined audit criteria during operative procedures. The audit tools captured compliance across all 27 criteria and included role-specific assessments for surgeons, assistant surgeons, scrub nurses, circulating nurses, anesthesiologists, and trainers.

Before data collection commenced, all observers received standardized orientation regarding the objectives of the audit, the operational definitions of each audit criterion, and the use of the structured audit checklist to promote consistent assessment and minimize observer variability. Observations were recorded contemporaneously during procedures to minimize recall bias.

Compliance was assessed at the level of individual observation opportunities. Consequently, the denominator for each criterion reflected the total number of eligible observations rather than the total number of surgical procedures. Criteria applicable to all operating theatre personnel generated a larger number of observations, whereas criteria specific to particular professional groups, procedural stages, or clinical circumstances generated fewer eligible observations. Criteria deemed not applicable to a specific staff member, procedure, or clinical situation were recorded as exceptions and excluded from the denominator for that criterion.

Outcome measures

The primary outcome measure was the compliance rate for each audit criterion, expressed as the proportion of observations meeting the predefined standard. Secondary outcome measures included: 1) Compliance rates within each major audit domain; 2) Compliance rates according to professional role; 3) Overall compliance across all assessed criteria; 4) Absolute percentage improvement between audit cycles; 5) Identification of persistent gaps requiring further quality improvement interventions.

Data analysis

Data were entered into Microsoft Excel (Microsoft Corp., Redmond, WA, USA) and analyzed using IBM SPSS Statistics for Windows, Version 25 (Released 2017; IBM Corp., Armonk, New York, United States). Descriptive statistics were used to summarize compliance rates across audit criteria, domains, and professional groups.

Categorical variables were presented as frequencies and percentages. Compliance rates were calculated using the number of compliant observations divided by the total number of eligible observations for each criterion. Because the applicability of individual criteria varied according to professional role and clinical circumstance, denominators differed between criteria. Comparisons between audit cycles were performed using Pearson’s Chi-square test or Fisher’s exact test when appropriate. Absolute percentage-point changes were calculated to quantify improvement following the intervention. Results were summarized using tables and graphical presentations to facilitate interpretation of trends and changes in compliance over time.

Compliance was evaluated at the level of individual observation opportunities because each audit criterion reflected the performance of specific operating theatre personnel and was not uniformly applicable across all staff members or procedures. Consequently, observations were analyzed at the criterion level. As multiple observations could arise from the same surgical procedure, potential clustering within procedures was not explicitly accounted for in the statistical analysis. Given the quality improvement nature of the audit and its primary objective of evaluating changes in compliance with predefined standards between audit cycles, this analytical approach was considered appropriate; however, the potential lack of independence between observations has been acknowledged as a study limitation.

Ethical considerations

This project was conducted as a quality improvement initiative aimed at enhancing patient safety and compliance with established infection prevention standards within the operating theatre. The project was thoroughly reviewed and approved by the Quality Improvement Committee of Atbara Teaching Hospital (approval no. 50/b/6) before commencement. The project was conducted in accordance with the hospital's ethical framework governing clinical governance and quality improvement activities.

No patient-identifiable information was collected at any stage of the project. All observations focused exclusively on clinical processes and professional practices. Data were anonymized prior to analysis, and confidentiality was maintained throughout the project. The project adhered to the principles of confidentiality, professional integrity, and accountability throughout all phases of implementation.

## Results

Compliance with surgical attire standards improved across all evaluated criteria between the first and second audit cycles (Table 1).

**Table 1:** Compliance with surgical attire standards before and after implementation of the quality improvement interventions n: number of compliant observations; N: total number of eligible observations. Compliance percentages were calculated using criterion-specific denominators. Denominators varied between criteria because not all observations were applicable to all professional categories or surgical situations. Pearson’s Chi-square test was used to compare compliance rates between the first and second audit cycles. Statistical significance was set at p<0.05.

Criterion	Cycle 1 n/N (%)	Cycle 2 n/N (%)	Absolute improvement (%)	χ²	p-value
Head cover covers all hair	83/174 (47.7)	106/150 (70.7)	+23.0	17.48	<0.001
Contaminated head cover changed when required	20/57 (35.1)	30/47 (63.8)	+28.7	8.53	0.004
Scrub suit donned in designated changing room	129/174 (74.1)	143/150 (95.3)	+21.2	26.86	<0.001
Inner clothing completely covered	96/174 (55.2)	126/150 (84.0)	+28.8	31.04	<0.001
Face mask completely covers nose and mouth	75/174 (43.1)	104/150 (69.3)	+26.2	22.42	<0.001

Compliance with appropriate head-cover use increased from 47.7% (83/174) to 70.7% (106/150; χ²=17.48; p<0.001). Compliance with replacement of contaminated head covers increased from 35.1% (20/57) to 63.8% (30/47; χ²=8.53; p=0.004). Adherence to changing into scrub suits within designated changing rooms improved from 74.1% (129/174) to 95.3% (143/150; χ²=26.86; p<0.001). Compliance with complete coverage of inner clothing increased from 55.2% (96/174) to 84.0% (126/150; χ²=31.04; p<0.001). Similarly, compliance with correct face-mask use increased from 43.1% (75/174) to 69.3% (104/150; χ²=22.42; p<0.001). Statistically significant improvements were observed for all assessed surgical attire criteria following implementation of the quality improvement interventions.

Compliance with surgical scrubbing technique standards improved across all assessed criteria between the first and second audit cycles (Table 2).

**Table 2:** Compliance with surgical scrubbing technique standards before and after implementation of the quality improvement interventions n: number of compliant observations; N: total number of eligible observations. Compliance percentages were calculated using criterion-specific denominators. Denominators varied according to the number of eligible observations for each criterion. Pearson’s Chi-square test was used to compare compliance between the first and second audit cycles. Statistical significance was set at p<0.05.

Criterion	Cycle 1 n/N (%)	Cycle 2 n/N (%)	Absolute Improvement (%)	χ²	p-value
Surgical scrub performed before gowning/gloving	30/60 (50.0)	60/68 (88.2)	+38.2	21.74	<0.001
Face mask secured prior to surgical scrub	37/108 (34.2)	78/109 (71.6)	+37.4	30.14	<0.001
Antimicrobial soap/alcohol-based antiseptic used	33/60 (55.0)	60/68 (88.2)	+33.2	16.94	<0.001
Fingernail hygiene maintained	63/110 (57.3)	91/114 (79.8)	+22.5	13.15	<0.001
Jewelry removed before surgical scrub	69/111 (62.4)	100/115 (87.0)	+24.6	18.18	<0.001
Correct surgical scrub technique used	21/60 (35.0)	47/68 (69.1)	+34.1	14.86	<0.001
Hands positioned above elbows after scrub	30/60 (50.0)	55/68 (80.9)	+30.9	13.43	<0.001
Correct drying technique before gowning	24/60 (40.0)	49/68 (72.1)	+32.1	13.29	<0.001

Compliance with performing surgical scrub before gowning or gloving increased from 50.0% (30/60) to 88.2% (60/68; χ²=21.74; p<0.001). Compliance with securing face masks prior to surgical scrub increased from 34.2% (37/108) to 71.6% (78/109; χ²=30.14; p<0.001), while appropriate use of antimicrobial soap or alcohol-based antiseptic increased from 55.0% (33/60) to 88.2% (60/68; χ²=16.94; p<0.001). Fingernail hygiene improved from 57.3% (63/110) to 79.8% (91/114; χ²=13.15; p<0.001), and compliance with jewelry removal increased from 62.4% (69/111) to 87.0% (100/115; χ²=18.18; p<0.001). Improvements were also observed in the use of correct surgical scrub technique, hand positioning following scrub, and drying technique before gowning, with all comparisons demonstrating statistically significant differences between audit cycles (p<0.001).

Compliance with gowning and gloving standards improved across all assessed criteria between the first and second audit cycles (Table 3).

**Table 3:** Compliance with gowning and gloving standards before and after implementation of the quality improvement interventions n: number of compliant observations; N: total number of eligible observations. Compliance percentages were calculated using criterion-specific denominators. Denominators varied according to the number of eligible observations for each criterion. Pearson’s Chi-square test was used for all comparisons except where expected cell counts were less than five. Statistical significance was defined as p<0.05.

Criterion	Cycle 1 n/N (%)	Cycle 2 n/N (%)	Absolute improvement (%)	χ²	p-value
Sterile gown donned before entering the sterile field	48/92 (52.2)	79/88 (89.8)	+37.7	31.45	<0.001
Sterile gown opened on a dedicated sterile surface	12/18 (66.7)	22/23 (95.7)	+29.0	5.57	0.018
Correct gowning steps followed	8/18 (44.4)	21/23 (91.3)	+46.9	10.13	0.001
Gown met quality standards	13/18 (72.2)	22/23 (95.7)	+23.5	4.47	0.035
Appropriate sterile glove application method used	13/18 (72.2)	23/23 (100.0)	+27.8	7.18	0.007

Compliance with sterile gown donning before entering the sterile field increased from 52.2% (48/92) to 89.8% (79/88; χ²=31.45; p<0.001). Compliance with opening sterile gowns on a dedicated sterile surface increased from 66.7% (12/18) to 95.7% (22/23; χ²=5.57; p=0.018). Adherence to the correct steps of gowning improved from 44.4% (8/18) to 91.3% (21/23; χ²=10.13; p=0.001). Compliance with gown quality standards increased from 72.2% (13/18) to 95.7% (22/23; χ²=4.47; p=0.035). Appropriate sterile glove application improved from 72.2% (13/18) to 100.0% (23/23; χ²=7.18; p=0.007). All assessed gowning and gloving criteria demonstrated statistically significant improvements following implementation of the quality improvement interventions.

Compliance with anaesthesia-related infection prevention practices improved significantly across all evaluated criteria between the first and second audit cycles (Table 4).

**Table 4:** Compliance with anaesthesia-related infection prevention practices before and after implementation of the quality improvement interventions n: number of compliant observations; N: total number of eligible observations. Compliance percentages were calculated using criterion-specific denominators. Denominators varied according to the number of eligible observations and exclusion of procedures classified as exceptions. Pearson’s Chi-square test was used for all comparisons except where expected cell counts were less than five. Statistical significance was defined as p<0.05. ^*^Exceptions (procedures in which no laryngoscope was used) were excluded from the denominator. ^†^Fisher’s exact test was used because of the presence of a zero cell.

Criterion	Cycle 1 n/N (%)	Cycle 2 n/N (%)	Absolute improvement (%)	χ²	p-value
Anesthesiologist glove use (single-use protocol)	6/18 (33.3)	18/23 (78.3)	+45.0	9.10	0.003
Anesthesiologist hand hygiene protocol	4/18 (22.2)	16/23 (69.6)	+47.4	10.14	0.001
Equipment cleaning after contamination	2/18 (11.1)	14/23 (60.9)	+49.8	11.99	<0.001
Glove use during intubation	8/18 (44.4)	20/23 (87.0)	+42.6	9.79	0.002
Laryngoscope sterilization between patients*	8/14 (57.1)	15/15 (100.0)	+42.9	8.04^†^	0.005

Compliance with anesthesiologist single-use glove protocol increased from 33.3% (6/18) to 78.3% (18/23; χ²=9.10; p=0.003). Compliance with anesthesiologist hand hygiene protocol increased from 22.2% (4/18) to 69.6% (16/23; χ²=10.14; p=0.001). Compliance with cleaning contaminated anesthetic equipment improved from 11.1% (2/18) to 60.9% (14/23; χ²=11.99; p<0.001). Appropriate glove use during intubation increased from 44.4% (8/18) to 87.0% (20/23; χ²=9.79, p=0.002). Compliance with laryngoscope sterilization between patients increased from 57.1% (8/14) to 100.0% (15/15; Fisher’s Exact Test, p=0.005). Statistically significant improvements were observed across all anaesthesia-related infection prevention practices following implementation of the quality improvement interventions.

Compliance with sterile supplies and operating room setup practices improved significantly across all assessed criteria between the first and second audit cycles (Table 5).

**Table 5:** Compliance with sterile supplies and operating room setup practices before and after implementation of the quality improvement interventions n: number of compliant observations; N: total number of eligible observations. Compliance percentages were calculated using criterion-specific denominators. Pearson’s Chi-square test was used to compare compliance between the first and second audit cycles. Statistical significance was set at p<0.05.

Criterion	Cycle 1 n/N (%)	Cycle 2 n/N (%)	Absolute Improvement (%)	χ²	p-value
Sterile supplies opened immediately before use	11/18 (61.1)	22/23 (95.7)	+34.6	8.12	0.004
OR furniture and equipment positioned before opening sterile supplies	10/18 (55.6)	22/23 (95.7)	+40.1	10.15	0.001
Sterile table positioned before opening sterile supplies	9/18 (50.0)	22/23 (95.7)	+45.7	12.12	<0.001
Correct sequence for opening sterile items followed	8/18 (44.4)	22/23 (95.7)	+51.3	14.26	<0.001

Compliance with opening sterile supplies immediately before use increased from 61.1% (11/18) to 95.7% (22/23; χ²=8.12; p=0.004). Compliance with positioning operating room furniture and equipment before opening sterile supplies increased from 55.6% (10/18) to 95.7% (22/23; χ²=10.15; p=0.001). Appropriate positioning of the sterile table improved from 50.0% (9/18) to 95.7% (22/23; χ²=12.12; p<0.001). Compliance with the correct sequence for opening sterile items increased from 44.4% (8/18) to 95.7% (22/23; χ²=14.26; p<0.001). Statistically significant improvements were observed across all sterile setup and supply management practices following implementation of the quality improvement interventions.

## Discussion

This closed-loop quality improvement project demonstrated substantial improvements in compliance with surgical scrubbing, sterilization, and infection prevention practices following implementation of a multifaceted intervention package. Significant improvements were observed across all assessed domains, including surgical attire, hand hygiene and surgical scrubbing techniques, gowning and gloving practices, anesthesia-related infection prevention measures, and maintenance of the sterile field. These findings suggest that structured audit cycles, targeted education, performance feedback, and multidisciplinary engagement were associated with improved adherence to evidence-based perioperative infection prevention standards.

The significant improvements observed in surgical attire practices are particularly noteworthy because operating room attire constitutes a fundamental component of aseptic practice and sterile field protection. Compliance improved significantly for head-cover use, face-mask application, replacement of contaminated head covers, and appropriate clothing coverage. International infection prevention guidance emphasizes that appropriate theatre attire, including complete hair coverage, proper mask use, and adherence to restricted-zone dress codes, contributes to reducing microbial dispersion within the operating room environment and supports maintenance of a sterile surgical field [9,10]. Although attire compliance alone cannot eliminate SSIs, consistent adherence remains an essential component of comprehensive perioperative infection prevention strategies.

Substantial improvements were also observed in surgical hand hygiene and scrubbing practices. Surgical hand antisepsis remains one of the most effective interventions for reducing transient and resident microbial flora prior to operative procedures and is widely recognized as a cornerstone of SSI prevention [8,11]. In the present audit, compliance improved significantly across multiple scrubbing-related criteria, including surgical hand preparation, use of antimicrobial agents, jewelry removal, hand positioning following scrubbing, and drying techniques. Similar findings have been reported in previous quality improvement studies demonstrating that educational interventions, direct observation, and structured feedback can significantly improve compliance with recommended surgical hand antisepsis protocols [12]. The magnitude of improvement observed in this study reinforces the value of continuous monitoring and targeted educational reinforcement in promoting safe perioperative practice.

The improvements observed in gowning and gloving practices were among the most marked findings of the audit. Compliance with correct gowning steps more than doubled, while appropriate sterile glove application achieved complete compliance during the re-audit cycle. These findings are consistent with previous clinical audits demonstrating that technical aseptic skills are highly responsive to audit-and-feedback interventions when deficiencies are identified and addressed through focused training programs [13]. Proper gowning and gloving represent critical barriers against microbial contamination and are integral to maintaining the integrity of the sterile field throughout operative procedures [8,14].

An important finding of the present study was the substantial improvement observed in anesthesia-related infection prevention practices. Baseline compliance among anesthesiology personnel was considerably lower than that observed among other professional groups, particularly regarding hand hygiene, glove use, and decontamination of contaminated equipment. Although significant improvements were achieved during the re-audit cycle, these criteria remained among the lowest-performing domains. Similar observations have been reported in perioperative infection prevention literature, where anesthesia-related infection control practices have historically received less attention than surgeon- and nurse-focused interventions despite the frequent contact of anesthesia personnel with airway devices, patient surfaces, monitoring equipment, and medication preparation areas [15]. Strengthening infection prevention awareness among anesthesia teams may therefore represent an important target for future quality improvement initiatives.

Significant improvements were likewise observed in sterile field preparation and management of sterile supplies. Compliance with appropriate timing of sterile supply opening, positioning of operating room equipment, sterile table preparation, and correct sequencing of sterile item opening increased markedly following intervention. These practices are fundamental components of aseptic technique and play an essential role in maintaining the sterility of surgical instruments and operative environments [10,16]. International perioperative guidelines consistently emphasize the importance of maintaining an intact sterile field and ensuring that all sterile items are handled according to standardized aseptic principles throughout the operative procedure [8,16].

The overall success of this project is likely attributable to the multifaceted nature of the intervention package. Educational sessions increased awareness of evidence-based standards, audit feedback highlighted specific performance gaps, visual reminders reinforced desired behaviors, and multidisciplinary engagement promoted collective responsibility for infection prevention. Previous quality improvement studies have demonstrated that audit-and-feedback interventions are particularly effective when combined with education, local ownership of improvement efforts, and repeated measurement of performance outcomes [17]. The findings of the present audit support the continued use of closed-loop audit methodology as an effective strategy for improving compliance with perioperative infection prevention standards in resource-limited healthcare settings.

Limitations

Several limitations should be acknowledged. This quality improvement project was conducted within a single institution, which may limit the generalizability of the findings to other healthcare settings. Additionally, the sample size was relatively small, and some audit criteria were applicable only to specific professional groups, resulting in variable denominators across assessments. The study evaluated compliance with surgical scrubbing, sterilization, and infection prevention practices rather than direct patient outcomes such as SSI rates. Furthermore, because operating theatre personnel were aware that observations were being conducted, a Hawthorne effect [18] may have contributed to some of the observed improvements during the re-audit cycle. Despite these limitations, the study provides valuable evidence that structured audit and targeted quality improvement interventions can substantially enhance adherence to evidence-based perioperative infection prevention standards.

## Conclusions

This closed-loop quality improvement project demonstrated that a multifaceted intervention consisting of education, audit feedback, visual reminders, and multidisciplinary engagement was associated with substantial improvements in compliance with evidence-based surgical scrubbing, sterilization, and infection prevention practices within the operating theatre. Improvements were observed across surgical attire, hand hygiene and surgical scrubbing techniques, gowning and gloving procedures, anesthesia-related infection prevention measures, and maintenance of the sterile field. These findings support the value of structured clinical audit and continuous quality improvement methodologies in improving adherence to perioperative infection prevention standards. Sustained monitoring, regular re-auditing, and ongoing staff education are recommended to maintain these improvements and may contribute to enhanced patient safety within surgical services.
